# Metabolic Profile of Patients with Premature Ovarian Insufficiency

**DOI:** 10.3390/jcm7100374

**Published:** 2018-10-21

**Authors:** Agnieszka Podfigurna, Angelika Stellmach, Anna Szeliga, Adam Czyzyk, Blazej Meczekalski

**Affiliations:** 1 Department of Gynecological Endocrinology, Poznan University of Medical Sciences, Polna 33, Poznan 60-535, Poland; agnieszkapodfigurna@gmail.com (A.P.); annamaria.szeliga@gmail.com (A.S.); czyzykadam@gmail.com (A.C.); 2 Students Scientific Society of the Department of Gynecological Endocrinology, Poznan University of Medical Sciences, Polna 33, Poznan 60-535, Poland; angelika.stellmach@gmail.com

**Keywords:** premature ovarian insufficiency, lipid profile, cardiovascular risk, insulin resistance

## Abstract

Premature ovarian insufficiency (POI) is hypogonadism associated with amenorrhea, increased levels of gonadotropins, and hypoestrogenism. Deficiency of estrogens may contribute to higher risk of cardiovascular diseases and death. POI patients present several risk factors for the development of cardiovascular diseases (CVD): endothelial dysfunction, abnormal lipid profile, insulin resistance, and insulin action disturbances. Therefore, patients present a higher risk of developing metabolic syndrome. Materials and methods: Follicle stimulating hormone (FSH), luteinizing hormone (LH), 17β-estradiol (E2), prolactin (PRL), testosterone (T), dehydroepiandrosterone sulfate (DHEA-S), thyroid stimulating hormone (TSH), thyroxine (fT4), fasting serum glucose and insulin concentrations, homeostatic model for insulin resistance (HOMA-IR), and lipid profiles were assessed in 56 women (mean age: 30.7 *±* 6.9) suffering from POI diagnosed according to European Society of Human Reproduction and Embryology (ESHRE) criteria and 68 healthy age-and-weight matched women (mean age: 27.3 *±* 4.5). Results: After regression analysis with BMI and age correction, total cholesterol (TC), high-density lipoprotein cholesterol (HDL-C), and low-density lipoprotein cholesterol (LDL-C) serum concentrations were found to be significantly higher in the POI group, when compared to healthy subjects, whilst triglycerides, glucose, insulin serum concentrations, HOMA-IR, as well as systolic (SBP) and diastolic blood pressure (DBP) did not differ significantly between both groups. A significant positive correlation was identified between TC and LDL-C levels, regardless of BMI and age, whilst SBP correlated only with serum glucose concentration. Additionally, FSH correlated positively with fasting serum glucose concentration after BMI and age correction. Conclusions: Certain metabolic parameters appeared to correlate with POI and these correlations persisted after correction for BMI and age. More research is required to determine the influence of absent ovulatory function on metabolic profiles in POI women. This information may additionally help in early identification of CVD risk factors in those patients.

## 1. Introduction

Premature ovarian insufficiency is defined as the loss of ovarian function before the age of 40 years. It is a state of female hypogonadism associated with amenorrhea, increased levels of gonadotropins, and hypoestrogenism [1,2]. The diagnostic criteria for POI require oligo/amenorrhea of at least four months duration and a follicle stimulation hormone (FSH) level above 25 IU/L, measured on two separate occasions at least four weeks apart [3]. The prevalence of POI in the general population is about 1%, and varies from 0.01% under the age of 20 years [4,5,6] to 1% of women below the age 40 years. POI can develop as a result of autoimmune, metabolic, or genetic factors, as well as secondary to infection (such as mumps virus) or iatrogenic causes (surgery, radiation, and chemotherapy) [7,8].

POI is a significant risk factor for health and disease susceptibility, including increased mortality [3,9]. It has been postulated that the loss of ovarian function and subsequent deficiency of endogenous estrogens in women with POI may contribute to higher risk of cardiovascular diseases (CVD) and death. POI patients often present several risk factors for the development of cardiovascular disease: Autonomic and endothelial dysfunction, abnormal lipid profile, and insulin dysfunction. As a result, they are at higher risk of metabolic syndrome development [9,10]. The mortality risk due to ischemic heart disease increases by approximately 80% in the POI group, when compared to women with menopause at 49–55 years [11]. To date, several studies have been performed to examine the metabolic profile in women affected by POI; however, results are conflicting [10].

The original intent of this study was to obtain additional data on the influence POI may have on women’s metabolic profile (lipid profile, glucose metabolism, and insulin resistance), and identification of possible biochemical or clinical indices which correlate with the severity of metabolic traits.

## 2. Materials

The study group included 56 women (mean age: 30.7 *±* 6.9; range: 16–40) who were newly diagnosed with POI according to the following criteria:

1. secondary amenorrhea of 4–7 months duration,
2. serum FSH concentration greater than 40 IU/L, measured on two separate occasions at least one month apart,
3. serum E2 concentration below 50 pg/mL [9,12,13],
4. idiopathic etiology of POI,

The control group consisted of 68 healthy age-and-weight matched women (mean age: 27.3 ± 4.5; range: 18–35). Inclusion criteria were:

1. Regular menstrual cycles (menses occurring at regular intervals of 27 to 35 days consecutively over the preceding six months per anamnesis).
2. No indication of hormonal abnormalities, as evidenced by screening hormone and metabolic profiles. (Table 1 and Table 2).
3. No indication of kidney or liver disease as evidenced by haematocrit, complete blood count, AST, ALT, and creatinine assessment, pelvic examination, and anamnesis.

Euovulatory state was confirmed by serum progesterone levels > 5 ng/mL in the luteal phase concurrent with the cycle at the time of examination.

The exclusion criteria for both groups were as follows:

1. Hormonal therapy at the time of examination or anytime in the preceding 6 months,
2. Severe chronic disease (with special emphasis on CVD),
3. Oncological treatment at any time in the patient’s history,

## 3. Methods

Body metrics were recorded for each patient, weight and height measurements were used to calculate BMI according to following formula: Weight (kg) divided by the square of height (m^2^). The international standard formula version served as the basis for calculating the Homeostatic Model Assessment-Insulin Resistance HOMA-IR: Fasting Glucose (mmol/L) × fasting Insulin (mU/L)/22.5. HOMA-IR > 2.5 indicated insulin resistance [13].

Each individual underwent transvaginal ultrasound examination with assessment of uterus and adnexa (Aloka Prosound Alpha 6, Hitachi Aloka, Tokyo, Japan). Blood pressure was measured using the auscultatory method and a sphygmomanometer. Venous blood samples were collected between 6.00 and 9.00 a.m. while in a fasting state. Blood samples from the control group were collected in the late follicular phase (10th–12th cycle day) and centrifuged at 1500 rpm to extract serum. We used the electrochemiluminescence immunoassay (Roche Diagnostics, Indianapolis, IN, USA) to determine the serum concentrations of: Follicle stimulating hormone (FSH), luteinizing hormone (LH), 17β-estradiol (E2), prolactin (PRL), testosterone (T), dehydroepiandrosterone sulfate (DHEA-S), thyroid stimulating hormone (TSH), thyroxine (fT4), fasting serum glucose and insulin concentrations, cholesterol (TC), high-density lipoprotein cholesterol (HDL-C), low-density lipoprotein cholesterol (LDL-C), and triglycerides (TG).

StatSoft 2012 STATISTICA Version 12 was used for statistical calculations. The Shapiro-Wilk test was performed to verify the normality of data distribution. The parametric Student’s *t*-test and the non-parametric Mann Whitney U tests were used to determine statistical significance. Correlation was determined using Spearman’s rank-order correlation, and regression analysis to correct for age and BMI was performed using the logistic model. A *p*-value of ≤0.05 was acknowledged as significant. The study protocol was examined and consent to proceed was granted by the supervising Bioethical Committee. Informed consent was obtained from all patients prior to sample collection.

## 4. Results

The baseline characteristics of both the POI and control group are presented in Table 1. As would be expected, patients in the POI group were found to have significantly higher FSH and LH serum concentrations and lower E2 serum levels, when compared to healthy controls. Additionally, there was a statistically significant difference in serum concentrations of T, DHEA-S, and PRL, with lower concentrations in the POI group. TSH and fT4 did not differ between the groups.

Women in the POI group had significantly higher serum TC and HDL-C concentrations when compared to healthy controls. Serum fasting concentration of LDL-C and TG did not differ between both groups. Fasting serum glucose and insulin concentrations, as well as HOMA-IR differed significantly between both groups (Table 2). Fasting serum glucose was found to be significantly higher in women with POI, whilst serum insulin concentration was lower. Diastolic blood pressure was significantly lower in the POI group when compared to healthy controls. However, it should be noted that initially the study and control groups were not matched for age and BMI. As both these factors have a significant impact on the above parameters, additional regression analysis was performed to correct for these variables. After correction using the logistic model of regression analysis, total cholesterol, HDL-C, and LDL-C serum concentrations were shown to be significantly higher in the POI group, when compared to healthy subjects. TG, glucose, and insulin serum concentrations, as well as HOMA-IR, did not differ between both groups. Moreover, after correcting for age and BMI, diastolic and systolic blood pressures no longer differed significantly between groups.

Abnormal HOMA-IR and fasting serum glucose concentrations were the most prevalent abnormalities in women with POI. HOMA-IR levels over 2.5 were found in 25% of POI patients, whilst fasting serum glucose >100 mg/dL was recorded in 21% of POI patients.

A noteworthy correlation was observed between certain elements of the hormone and metabolic profiles of both the studied and control groups (Table 3). Blood pressure correlated positively with certain lipid fractions and metabolic parameters. In the POI group, SBP correlated positively with LDL-C, insulin, and HOMA-IR, whilst DBP correlated positively with LDL-C, TC, insulin, and HOMA-IR. However, these results were not controlled for age and BMI. As both these variables can influence metabolic parameters, additional regression analysis was performed to correct for these variables. Following this additional analysis, DBP continued to correlate positively with TC and LDL-C, whilst SBP correlated only with serum glucose concentration. Additionally, FSH correlated positively with fasting serum glucose concentration both before and after correction for BMI and age.

## 5. Discussion

It has been proposed that since POI is associated with the symptoms of early menopause, it may also lead to abnormal lipids metabolism, impaired glucose tolerance, and elevated cardiovascular risk. The present study was conducted to assess the metabolic consequences of POI, and to compare the metabolic profile of POI patients with that of age-matched premenopausal healthy controls. The current literature is inconsistent when considering the relationship between premature menopause and lipid or glucose metabolism. In the present study, we reported higher serum concentrations of TC, LDL-C, and HDL-C in the POI group, when compared to healthy controls.

In the 1990s, while studying CVD related deaths in Norwegian cohorts, Jacobsen et al. found a modest inverse relationship between cardiovascular disease mortality and age at menopause [14]. Similar conclusions were drawn by Var der Schouw et al. in a Dutch cohort, and again by Jacobsen et al. in an American Seventh-Day-Adventist cohort [10,15,16].

In the first study of its kind, Kalantaridou et al. examined the metabolic panel of 18 patients with POI, specifically fasting serum glucose and lipoproteins. They concluded that no significant difference could be observed when compared with 20 healthy controls [11]. In a later trial, scientists detected higher concentrations of TG and significantly lower HDL-C in POI patients when compared to controls [17]. LDL-C and TC were found to be similar in both groups. The authors of the latter study suggested that such a lipid profile may be due to relative androgen excess or insulin resistance. Nevertheless, subsequent trials involving POI patients revealed an overall decrease in circulating androgen concentrations, subsequent dyslipidemia, and an increased risk of cardiovascular disease [18]. In the current trial, significantly lower serum concentrations of T and DHEA-S were observed in POI patients; however, no significant correlation could be drawn between androgen and lipid levels either before or after correction for BMI.

Gulhan et al. [19] compared 407 POI women with 60 age-and-weight matched controls. They revealed a statistically significant increase in TC and LDL-C concentration in the POI group, when compared to controls. They also found a higher serum HDL-C concentration in women suffering from POI [19], a finding we confirmed in the present study. Several studies have examined how HDL-C levels change after menopause and as women transition into menopause. However, results from these trials are inconsistent and conflicting [20,21,22]. Certain studies show that HDL-C does not significantly fluctuate or can even increase [22], whereas in others they show a decrease in concentration of HDL-C after the onset of natural menopause [21].

The results we presented are also partially consistent with those published by Ates et al., where HDL-C and TC levels were higher in POI women when compared to controls, whilst LDL-C and TG levels did not differ between groups [23]. In its current state, the literature stops short of providing any adequate explanation of the mechanism behind this finding. Certain authors postulate that the elevation in HDL-C occurs as a result of unspecified hormonal changes secondary to ovarian insufficiency. Nevertheless, most studies to date (this study included) lack strict control of external factors influencing a patient’s HDL-C and lipid profile, such as physical activity or diet.

When considering glucose metabolism, Ates et al. did not report any significant difference in insulin sensitivity between POI patients and healthy controls, nor did they report abnormal glucose and insulin fasting serum concentrations [23]. In contrast, Kulaksizoglu et al. [24] described increases in serum glucose and insulin concentrations, resulting in abnormal HOMA-IR in POI patients. Corrigan et al. also reported decreased insulin sensitivity in POI patients when compared to healthy, but more obese controls [25]. In the present study, fasting serum glucose and insulin concentrations did not differ significantly once groups were adjusted for BMI and age, confirming the results reported earlier by Ates et al. [23]. It should be noted, that when compared to the patient sample used by Ates et al., this study counted a higher overall percentage of women with POI having abnormal fasting glucose concentrations (21% vs. 9%), but with a lower percentage of these patients suffering from insulin resistance (25% vs. 37%). Moreover, it was found that HOMA-IR values correlated positively with BMI in healthy controls, but not in the POI group.

When considering the current literature on the effects of POI on lipid and glucose metabolism, the variability of results can largely be attributed to the consistently small sample sizes studied. Such small sample sizes are inadequate for producing accurate results. Naturally, the next step in the progression of assessing the influence of POI on women’s health is undertaking statistically robust, large-scale studies. Moreover, undertaking a larger study would allow for the consideration and control of additional factors of influence on glucose and cholesterol metabolism, such as diet and physical activity.

## 6. Conclusions

This study demonstrates that high TC, LDL-C, and HDL-C serum levels may be related to premature ovarian insufficiency and that patients with POI have normal TG levels. We have found no difference in fasting serum glucose concentration, serum insulin concentration, and HOMA-IR of women with POI when compared to controls. Certain metabolic parameters seem to correlate strongly with POI, even when corrected for BMI and age. Further research is required to determine the extent to which the absence of ovulatory function affects metabolic profiles in POI women. A further understanding may help in the early identification of CVD risk factors in these patients.

## Figures and Tables

**Table 1:** Demographic, clinical, and hormonal parameters of POI and control groups. Data shown as mean ± SD; ^a^ Student’s *t*-test; ^b^ Mann-Whitney U test (*p*-values < 0.05 are bolded). BMI—body mass index; FSH—follicle stimulating hormone; LH—luteinizing hormone; E2—17β-estradiol; PRL—prolactin, T—testosterone; DHEA-S—dehydroepiandrosterone sulfate; TSH—thyroid stimulating hormone; fT4—thyroxine.

	POI Women (*n* = 56)	Controls (*n* = 68)	*p*-Value
**Age** (year) ^b^	30.7 ± 6.9	27.3 ± 4.5	**<0.001**
**BMI** (kg/m^2^) ^b^	23.54 ± 4.55	28.62 ± 5.30	**<0.001**
**FSH** (U/L) ^b^	97.57 ± 42.30	5.82 ± 2.19	**<0.001**
**LH** (U/L) ^b^	50.93 ± 26.62	13.88 ± 9.68	**<0.001**
**E2** (pg/mL) ^b^	21.45 ± 43.07	172.64 ± 134.71	**<0.001**
**T** (ng/mL) ^b^	0.27 ± 0.14	0.55 ± 0.20	**<0.001**
**PRL** (ng/mL) ^b^	10.08 ± 4.83	17.26 ± 9.18	**<0.001**
**DHEA-S** (umol/L) ^a^	6.54 ± 2.86	7.83 ± 3.30	**0.046**
**TSH** (uIU/mL) ^b^	2.46 ± 1.53	2.31 ± 1.04	0.93
**fT4** (ng/dL) ^a^	1.32 ± 0.22	1.29 ± 0.17	0.34

**Table 2:** Metabolic parameters in women with POI and healthy controls. Data shown as mean ± SD; ^a^ Student’s *t*-test; ^b^ Mann-Whitney U test (*p*-values <0.05 are bolded). TC—total cholesterol; HDL-C—high-density lipoprotein cholesterol; LDL-C—low-density lipoprotein cholesterol; TG—triglycerides; HOMA-IR—Homeostatic Model Assessment-Insulin Resistance; SBP—systolic blood pressure; DBP—diastolic blood pressure.

	POI Group (*n* = 56)	Controls (*n* = 68)	*p*-Value	*p*-Value	95% OR
				After BMI and Age Correction	
**TC** [mg/dL] ^b^	203.01 ± 32.38	163.74 ± 16.97	**<0.0001**	**<0.001**	1.053–1.138
**HDL-C** [mg/dL] ^b^	74.03 ± 18.73	57.00 ± 14.18	**<0.0001**	**0.002**	1.017–1.077
**LDL-C** [mg/dL] ^b^	114.70 ± 31.17	91.65 ± 15.18	0.71	**<0.001**	1.042–1.119
**TG** [mg/dL] ^b^	77.26 ± 48.47	76.08 ± 26.85	0.33	0.54	-
**Glucose** [mg/dL] ^b^	91.87 ± 8.40	88.40 ± 22.98	**0.0003**	0.85	-
**Insulin** [mU/mL] ^b^	9.28 ± 6.45	11.62 ± 5.52	**0.002**	0.82	-
**HOMA-IR** ^b^	2.09 ± 1.70	2.50 ± 1.36	**0.03**	0.66	-
**SBP** [mmHga] ^b^	113 ± 11	117 ± 9	0.08	0.62	-
**DBP** [mmHga] ^b^	70 ± 8	75 ± 7	**0.004**	0.63	-

**Table 3:** Correlations between hormone and metabolic parameters of POI patients. Data shown as correlation coefficient (R) before and after regression analysis (R shown only if *p*-values < 0.05; *p*-values > 0.05 marked as non-significant [ns]). BMI—body mass index; TC—total cholesterol; HDL-C—high-density lipoprotein cholesterol; LDL-C—low-density lipoprotein cholesterol; TG—triglycerides; HOMA-IR—Homeostatic Model Assessment-Insulin Resistance; SBP—systolic blood pressure; DBP—diastolic blood pressure; FSH—follicle stimulating hormone; LH—luteinizing hormone; E2—17β-estradiol; T—testosterone; DHEA-S—dehydroepiandrosterone sulfate;

POI Group								
		SBP	DBP	FSH	LH	E2	T	DHEA-S
BMI	R	**0.30**	**0.39**	**−0.32**	ns	**0.35**	ns	ns
Glucose	R	ns	ns	**0.33**	ns	ns	ns	ns
	R after BMI and age correction	**0.37**	ns	**0.40**	ns	ns	ns	ns
Insulin	R	**0.34**	**0.30**	ns	ns	ns	ns	ns
	R after BMI and age correction	ns	ns	ns	ns	ns	ns	ns
HOMA-IR	R	**0.34**	**0.29**	ns	ns	ns	ns	ns
	R after BMI and age correction	ns	ns	ns	ns	ns	ns	ns
TC	R	ns	**0.30**	ns	ns	ns	ns	ns
	R after BMI and age correction	ns	**0.30**	ns	ns	ns	ns	ns
TG	R	ns	ns	ns	ns	ns	ns	ns
	R after BMI and age correction	ns	ns	ns	ns	ns	ns	ns
LDL-C	R	**0.27**	**0.39**	ns	ns	ns	ns	ns
	R after BMI and age correction	ns	**0.37**	ns	ns	ns	ns	ns
HDL-C	R	ns	ns	ns	ns	ns	ns	ns
	R after BMI and age correction	ns	ns	ns	ns	ns	ns	ns
